# Differential mRNA Expression in Ileal Mucosal Biopsies of Patients With Diarrhea- or Constipation-Predominant Irritable Bowel Syndrome

**DOI:** 10.14309/ctg.0000000000000329

**Published:** 2021-04-12

**Authors:** Xiao Jing Wang, Paula Carlson, Victor Chedid, Daniel B. Maselli, Ann L. Taylor, Sanna McKinzie, Michael Camilleri

**Affiliations:** 1 Clinical Enteric Neuroscience Translational and Epidemiological Research (CENTER), Mayo Clinic, Rochester, Minnesota, USA.

## Abstract

Previous studies in patients with irritable bowel syndrome (IBS) showed immune activation, secretion, and barrier dysfunction in duodenal, jejunal, or colorectal mucosa. This study aimed to measure ileal mucosal expression of genes and proteins associated with mucosal functions.

**METHODS:**

We measured by reverse transcription polymerase chain reaction messenger RNA (mRNA) expression of 78 genes (reflecting tight junction proteins, chemokines, innate immunity, ion channels, and transmitters) and 5 proteins (barrier, bile acid receptor, and ion exchanger) in terminal ileal mucosa from 11 patients with IBS-diarrhea (IBS-D), 17 patients with IBS-constipation (IBS-C), and 14 healthy controls. Fold changes in mRNA were calculated using 2^(−Δ, ΔCT)^ formula. Group differences were measured using analysis of variance. Protein ratios relative to healthy controls were based on Western blot analysis. Nominal *P* values (*P* < 0.05) are reported.

**RESULTS:**

In ileal mucosal biopsies, significant differences of mRNA expression in IBS-D relative to IBS-C were upregulation of barrier proteins (TJP1, FN1, CLDN1, and CLDN12), repair function (TFF1), and cellular functions. In ileal mucosal biopsies, mRNA expression in IBS-C relative to healthy controls was reduced GPBAR1 receptor, myosin light chain kinase (MYLK in barrier function), and innate immunity (TLR3), but increased mRNA expression of cadherin cell adhesion mechanisms (CTNNB1) and transport genes SLC9A1 (Na-H exchanger [NHE1]) and INADL (indirect effect on ion transport).

**DISCUSSION:**

These data support a role of ileal mucosal dysfunction in IBS, including barrier dysfunction in IBS-D and alterations in absorption/secretion mechanisms in IBS-C.

## INTRODUCTION

Irritable bowel syndrome (IBS) is considered to be a multifactorial disease with several central mechanisms (1), and peripheral mechanisms associated with organ-level dysfunctions including bowel hypersensitivity, altered motility, inflammation, enteroendocrine cell products, and effects of intraluminal content including bile acids, short-chain fatty acids and microbiota; mucosal barrier proteins; mast cell products such as histamine, proteases, and tryptase; enteroendocrine cell products; and mucosal messenger RNAs (mRNAs), proteins, and microRNAs (2–5). Intestinal secretion (6) is associated with several biomarkers: increased duodenal and rectosigmoid expression of secretory transmitters (e.g., serotonin [5-HT]), reduced expression of the serotonin reuptake protein (7,8), or reduced mucosal expression of proabsorption mechanisms (e.g., peptide YY [PYY] and somatostatin, neuropeptide Y) (7). Moreover, increased fecal excretion of secretogranins or chromogranins accelerates colonic transit (9).

Irritable bowel syndrome has also been associated with changes in rectosigmoid mucosal mRNA expression of immune factors, barrier function, and mucus secretion (10–16). The upregulated mechanisms measured initially by RNA sequencing in 9 patients with diarrhea-predominant IBS (IBS-D) were associated with changes in ion transport and included PDZ domain-containing 3 (PDZD3) and guanylate cyclase activator 2B (GUCA2B) (14). These results in rectosigmoid mucosal biopsies were largely replicated using reverse transcription polymerase chain reaction in 47 patients with IBS-D (17). PDZ adapter proteins are involved in multiple ion transport functions in the intestine, and *GUCA2B* is the gene controlling endogenous uroguanylin which induces chloride secretion. Additional abnormal mRNA expression in rectosigmoid mucosal biopsies included mRNA of receptors or neurotransmitters (P2RY4 and vasoactive intestinal peptide), cytokines and complement components (C4BPA and CCL20), immune function (e.g., TNFSF15), and mucosal repair and cell adhesion (TFF1 and FN1).

Studies reported by Martinez et al. (18,19) document in patients with IBS-D differences in jejunal mucosal expression (at gene and protein levels) of apical junction complex proteins, consistent with altered barrier mechanisms and mast cell activation. Similarly, a pilot study of duodenal mucosal biopsies from 15 patients with IBS-D also showed upregulation (uncorrected *P* < 0.05) in mRNA expression of genes related to ion transport (INADL), barrier function (TJP1,2, and 3 and CLDN), immune functions (TLR3, IL15, and MAPKAPK5), or histamine metabolism (HNMT); by contrast, other genes related to immune function (IL-1β, TGF-β1, and CCL20) or antigen detection (TLR1 and 8) were downregulated (20).

Less is known about the functions of the ileal mucosa in patients with IBS with either constipation or diarrhea. Yet, ileal mucosal function is relevant in diarrheal diseases; e.g., postvagotomy diarrhea is associated with increased serosa-to-mucosa flux as well as increased interepithelial cell leakiness (21). Ileal bile acid activation of nuclear farnesoid X receptor and mucosal expression of FGF19 mRNA are lower in patients with primary bile acid diarrhea (22,23). The mucosal functions of the ileum of patients with IBS are unclear.

In this study, our aim was to compare mucosal gene expression in terminal ileal mucosal biopsies of patients with IBS-D or constipation-predominant IBS (IBS-C) and healthy controls. To achieve this goal, we examined the expressions of 78 genes potentially associated with the pathobiology of IBS including tight junction proteins, chemokines and other markers of inflammation, innate immunity, receptor function, and ion channels associated with absorption and secretion.

## METHODS

### Ethical approval

The study was approved by Mayo Clinic Institutional Review Board on May 20, 2016 (IRB #16-001445). Written informed consent was received from participants before inclusion in the study.

### Study design

We measured mRNA expression in mucosal biopsies from the terminal ileum in 11 patients with IBS-D, 17 patients with IBS-C, and 14 healthy controls and quantitated 5 selected proteins in mucosal biopsies from 10 IBS-D, 10 IBS-C, and 10 healthy controls.

### Patient selection

Patients were recruited by public advertisement or by invitation to participate from a database of ∼1,200 patients with IBS living within ∼120 miles of Mayo Clinic in Rochester, MN. Inclusion criteria were based on symptoms using a validated diary questionnaire that characterized IBS symptoms and particularly bowel functions (24) and were consistent with Rome III criteria for IBS (25) which were the relevant criteria at the time of protocol approval (DK115950). Patients were screened using an initial 1 page evaluation of gastrointestinal symptoms, followed by the validated bowel disease questionnaire (24) at time of study enrollment. This was performed to characterize IBS phenotype and ensure consistency in IBS and bowel function phenotype with that stated in patient's medical record. Patients with mixed-type IBS or fluctuating phenotypes were not included in the study. These patients had been evaluated at Mayo Clinic, and alternative diagnoses such as inflammatory bowel disease, cancer, celiac disease, any autoimmune disorders, and any other systemic illness that were not under control were excluded. A list of medications taken by patients is included in Supplementary Table 2 (see Supplementary Digital Content 1, http://links.lww.com/CTG/A534). A list of reported patient allergies is provided in Supplementary Table 3 (see Supplementary Digital Content 1, http://links.lww.com/CTG/A534). Patient enrollment for the study began in June 2018, and the participation of the cohort in this study was completed in July 2020.

### Patient and public involvement

These studies were performed as part of an NIH-funded grant R01-DK115950 (Federal Award Date: December 27, 2017). Therefore, they were peer reviewed by a body of scientists and clinicians appointed by the National Institutes of Health and started in January 2018. The human studies were peer reviewed at the end of 2017 and first quarter of 2018 by the Mayo Clinic Institutional Review Board, which includes members of the lay public. These peer review entities assessed the research questions and outcome measures based on the significance of the questions (NIH review) and relevance/merit for the general public (NIH and IRB). Patients and the public were not directly involved in the design of the study; however, they were indirectly involved by virtue of previous interactions during preliminary studies conducted by the principal investigator who informed the priorities and design of the study.

Recruitment to the study was conducted by public advertisements available for review by patients and employees at Mayo Clinic, Rochester, MN. Patients with previous authorization for review of their medical record and previous diagnoses of relevance to this research were invited to participate using information and script that was approved by the Mayo Clinic IRB. Patients were given opportunity to voice any concerns at exit interviews with the study coordinators and investigators. The results of research studies are communicated to any participant that requests further information and is submitted for consideration of dissemination in the Mayo Clinic Health Letter.

### Tissue acquisition

Before the day of endoscopy and biopsy acquisition, patients were asked to discontinue use for at least 72 hours of any medications that may impact bowel function. Patient underwent bowel preparation with GoLYTELY (Braintree Laboratories, Braintree, MA), and underwent full colonoscopy under conscious sedation with midazolam, and analgesia with fentanyl and continuous monitoring of vital signs and oxygen saturation. Examination included terminal ileum intubation and biopsy.

### Selection of genes of interest

We developed a custom profile including 78 genes (reflecting tight junction proteins, chemokines, innate immunity, ion channels, and transmitters; see Supplementary Materials Table 1, Supplementary Digital Content 1, http://links.lww.com/CTG/A534), extending from the previous 19 gene profile used in a study of rectosigmoid mucosa (14). In addition, we included controls to monitor for DNA contamination and first strand synthesis and polymerase chain reaction efficiency to check for sample quality and reaction quality. The methods used followed those used in our laboratory in a previous study of duodenal mucosa in IBS-D (20).

Details on acquisition and storage of biospecimens, mRNA extraction, Gene Expression Method by RT2 PCR Array focused on 78 genes whose general functions are included in Supplementary Materials Table 1 (see Supplementary Digital Content 1, http://links.lww.com/CTG/A534). Protein measurements by Western blot, pathway and cluster analysis based on lens for enrichment and network studies of proteins, and analysis of gene expression and overlap of pathways using gene set enrichment analysis (26–28) are provided in the Supplementary Materials (see Supplementary Digital Content 1, http://links.lww.com/CTG/A534).

### Data analyses

#### mRNA expression.

Calculation of the threshold cycle (C_T_) was determined for each well. Briefly, using the ViiA7 Software on the real-time machine, baseline was defined by choosing the automated baseline option, and threshold was defined manually using the log view of the amplification plots. The C_T_ value was chosen above the background signal, but within the lower one-third to lower one-half of the linear phase of the amplification plot. In our study, the threshold was chosen at 0.16 for all plates. C_T_ values for all wells were exported to Excel and analyzed.

Delta C_T_ (ΔC_T_) was calculated between mRNA expression of gene of interest and an average of housekeeping genes (ACTB and GAPDH). A lower ΔC_T_ relative to the housekeeping gene(s) reflects higher expression compared with a numerically higher ΔC_T_.

#### Protein quantification.

##### Proteins of interest.

To explore whether the significant mRNA gene expression with greatest potential mechanistic relevance translated in resulting protein, quantification by Western blot was performed on 10 samples per participant group. Thus, based on mRNA gene expression of IBS-C or IBS-D compared with health, the statistically significant genes that represented important mechanisms of interest were TJP1, OCLN, CLDN1 (all related to barrier function), GPBAR1 (related to effector mechanism for bile acids), and SLC9A1 (related to epithelial transport), with housekeeping genes ACTB and GAPDH. The 10 biopsy samples in each group were chosen based on the amount of sample material available.

Western blots were scanned and quantified by ImageJ, and each lane was normalized to β-actin. The normalized protein levels were compared directly for the 3 groups using analysis of variance on ranks. In addition, to control for potential differences between the blots, IBS-C and IBS-D samples were expressed as a ratio relative to mean for healthy controls within each blot.

### Statistical analysis

Statistical analyses were conducted using SigmaPlot 12 software (Copyright 2011–2012 Systat Software). We compared the ΔC_T_ values for the 3 groups by ANOVA, or ANOVA on ranks when the data were nonparametric; when the 3-group comparison was significant, pairwise comparisons (Tukey test) addressed differences between IBS-C compared with healthy controls, and IBS-D compared with IBS-C as disease control. When the ANOVA *P* value was <0.25, we pursued the same 2-group comparisons (health vs IBS-C, and IBS-D vs IBS-C) using the nonpaired *t* test or Mann-Whitney *U* test in accordance with normal or nonparametric distribution of data, respectively. The *P* values (*P* < 0.05) were reported based on statistical analysis tests without false detection rate (FDR) correction (29). The data were then appraised for the direction of statistically different levels of expression between IBS-C and health, and between IBS-D compared with IBS-C, noting that a lower ΔC_T_ relative to the housekeeping gene(s) reflects higher expression compared with a numerically higher ΔC_T_.

Data on mRNA expression in the literature are often reported as fold change and fold regulation, according to the following calculations:

Fold change was calculated as the ratio between groups of interest by the 2^(−Δ, ΔCT)^ formula: Fold change = 2^-(ΔCT test group)/2^-(Δ CT control group).

Fold regulation was calculated by the formula: −(1/fold change), if fold change <1. If the fold change is >1, the fold change and fold regulation are equal.

## RESULTS

### Patients

We studied 11 patients with IBS-D, 17 patients with IBS-C (Rome III positive), and 14 healthy controls (Table 1). Participant demographics and measurements of colonic transit and bile acid parameters available are included in Table 1. As expected, there were significant differences in colonic transit at 24 and 48 hours between IBS-D and IBS-C (Table 1).

**Table 1.** Patient demographics and measurements of colonic transit and bile acid parameters available

	Healthy (N = 14)	IBS-C (N = 17)	IBS-D (N = 11)	*P*^a^
Female sex, n (%)	6 (42.9)	17 (100)	10 (90.9)	
Age, yr (SE)	47 (3.2)	52 (2.9)	48.3 (3.6)	
Race, n (%)				
White	14 (100)	17 (100)	9 (81.8)	
Black or African American			1 (9.1)	
Other			1 (9.1)	
Colonic transit, median (IQR)	N = 5	N = 12	N = 9	
24 hr	2.28 (1.43–4.2)	1.72 (1.52–1.87)	3.35 (2.37–4.30)	<0.001
48 hr		2.69 (2.25–3.06)	5.0 (4.83–5.0)	0.003
48h fecal bile acids, median (IQR)	N = 4		N = 5	
% CDCA+CA	0.7 (7.3)	ND	1.7 (20)	
Total	1,063 (1,611)		2,199 (2,138)	
Serum 7aC4	N = 13	N = 15	N = 11	
Mean (SD)	15.5 (11.2)	22.5 (24.2)	39.4 (33.7)	
Median (IQR)	14.8 (16.9)	6.4 (35.7)	27.7 (64.5)	

7aC4, 7 alpha-hydroxy-4-cholesten-3-one; CA, cholic acid; CDCA, chenodeoxycholic acid; IBS-C, constipation-predominant irritable bowel syndrome; IBS-D, diarrhea-predominant irritable bowel syndrome; IQR, interquartile range; ND, not done.

a *P* value based on the Mann-Whitney rank-sum test for IBS-D compared with IBS-C groups.

### mRNA in ileal mucosa from IBS-C, IBS-D, and healthy controls

Table 2 provides the detailed information regarding ΔC_T_ of 24 genes that showed either overall statistical significance or pairwise comparisons (IBS-C vs health and IBS-C vs IBS-D) with *P* values <0.10. In these ileal mucosal biopsies, comparison of mRNA expression among the 3 groups showed significant differences, controlled for housekeeper genes.

**Table 2.** ΔC_T_ expression of mRNA in ileal biopsies of HC, and IBS-C and IBS-D patients; a lower ΔC_T_ between 2 groups implies higher expression

Symbol	ΔC_T_ health	ΔC_T_ IBS-C	ΔC_T_ IBS-D	*P* ANOVA	*P* health vs IBS-C	*P* health vs IBS-D	*P* IBS-C vs IBS-D	Expression IBS-C vs HC IBS-D vs IBS-C
Cell adhesion, tight junctions, or barrier function								
TFF1	4.29 ± 0.20	5.08 ± 0.18	4.00 ± 0.28	0.002	0.022	0.645	0.004	↓IBS-C; ↑IBS-D
FN1	7.25 ± 0.21	7.73 ± 0.18	6.94 ± 0.21	0.029	0.096	0.320	0.027	↑ IBS-D
CLDN1	11.10 ± 0.30	11.82 ± 0.28	10.71 ± 0.43	0.063	0.096	0.452	0.033	↑ IBS-D
CLDN12	7.15 ± 0.16	7.53 ± 0.15	7.03 ± 0.16	0.066	0.088	0.620	0.035	↑ IBS-D
PVRL3 (Nectin3)	5.12 ± 0.14	5.45 ± 0.10	5.03 ± 0.14	0.049	0.134	0.895	0.068	
OCLN	5.02 ± 0.16	5.21 ± 0.14	4.80 ± 0.21	0.241	0.38	0.403	0.060	
MYLK	5.44 ± 0.16	5.82 ± 0.16	5.37 ± 0.18	0.124	0.022	0.786	0.087	↓ IBS-C
TJP1	6.10 ± 0.17	6.26 ± 0.07	5.75 ± 0.16	0.034	0.643	0.187	0.027	↑ IBS-D
FGFR4	10.95 ± 0.20	10.87 ± 0.11	10.41 ± 0.31	0.127	ns	0.097	0.099	
Neurotransmitters, exchangers, and receptors								
GPBAR1	11.74 ± 0.16	12.31 ± 0.14	11.95 ± 0.17	0.032	0.027	0.657	0.258	↓ IBS-C
HNMT	10.05 ± 0.24	10.59 ± 0.16	10.08 ± 0.28	0.133	0.066	0.919	0.114	
HRH1	8.20 ± 0.12	8.30 ± 0.11	8.02 ± 0.24	0.196	0.539	0.483	0.09	
HRH2	11.50 ± 0.32	12.14 ± 0.59	10.96 ± 0.33	0.205	0.677	0.251	0.067	
SLC9A1	8.12 ± 0.12	7.75 ± 0.10	7.71 ± 0.36	0.089	0.020	0.529	0.54	↑ IBS-C
KITLG	6.63 ± 0.17	7.03 ± 0.13	6.29 ± 0.13	0.005	0.120	0.301	0.004	↑ IBS-D
INADL (PATJ)	7.25 ± 0.10	6.92 ± 0.09	7.11 ± 0.29	0.157	0.018	0.494	0.888	↑ IBS-C
SOS1	6.18 ± 0.17	6.31 ± 0.07	5.98 ± 0.14	0.230	0.470	0.402	0.034	↑ IBS-D
Cell kinetics								
CTNNB1	6.58 ± 0.11	6.26 ± 0.09	6.47 ± 0.24	0.185	0.033	0.338	0.707	↑ IBS-C
CALR	3.81 ± 0.16	4.10 ± 0.07	3.46 ± 0.11	0.002	0.162	0.107	0.001	↑ IBS-D
PPP1CB	2.69 ± 0.10	2.83 ± 0.04	2.61 ± 0.07	0.099	0.117	0.518	0.008	↑ IBS-D
Innate immunity and inflammation								
TLR1	10.51 ± 0.34	11.17 ± 0.31	10.20 ± 0.50	0.237	0.162	0.891	0.094	
TLR3	6.60 ± 0.13	7.07 ± 0.18	6.83 ± 0.22	0.143	0.050	0.347	0.407	↓ IBS-C
TLR5	10.76 ± 0.24	11.14 ± 0.20	10.57 ± 0.23	0.191	0.238	0.580	0.079	
IL15	8.67 ± 0.16	8.98 ± 0.12	8.62 ± 0.14	0.139	0.117	0.835	0.068	

CALR, calreticulin; CLDN1, claudin-1; CLDN12, claudin-12; CTNNB1, catenin beta 1; FGFR4, fibroblast growth factor receptor 4; FN1, fibronectin 1; GPBAR1, G-protein–coupled bile acid receptor 1; HC, healthy controls; HNMT, histamine n-methyl transferase; HRH1, histamine receptor H1; HRH2, histamine receptor H2; IBS-C, constipation-predominant irritable bowel syndrome; IBS-D, diarrhea-predominant irritable bowel syndrome; IL15, interleukin 15; INADL, inactivation-no-afterpotential D-like (also known as PATJ); KITLG, KIT ligand; mRNA, messenger RNA; MYLK, myosin light chain kinase; OCLN, occludin; PPP1CB, protein phosphatase 1 catalytic subunit beta; PVRL3, poliovirus-receptor-related 3 (also known as nectin 3); SLC9A1, solute carrier family 9 member A1; SOS1, Sons of Sevenless (*Drosophila*) homolog 1; TFF1, trefoil factor 1; TJP1, tight junction protein 1; TLR1, toll-like receptor 1; TLR3, toll-like receptor 3; TLR5, toll-like receptor 5; ΔC_T_, change in calculation of the threshold cycle.

**Figure 1.**
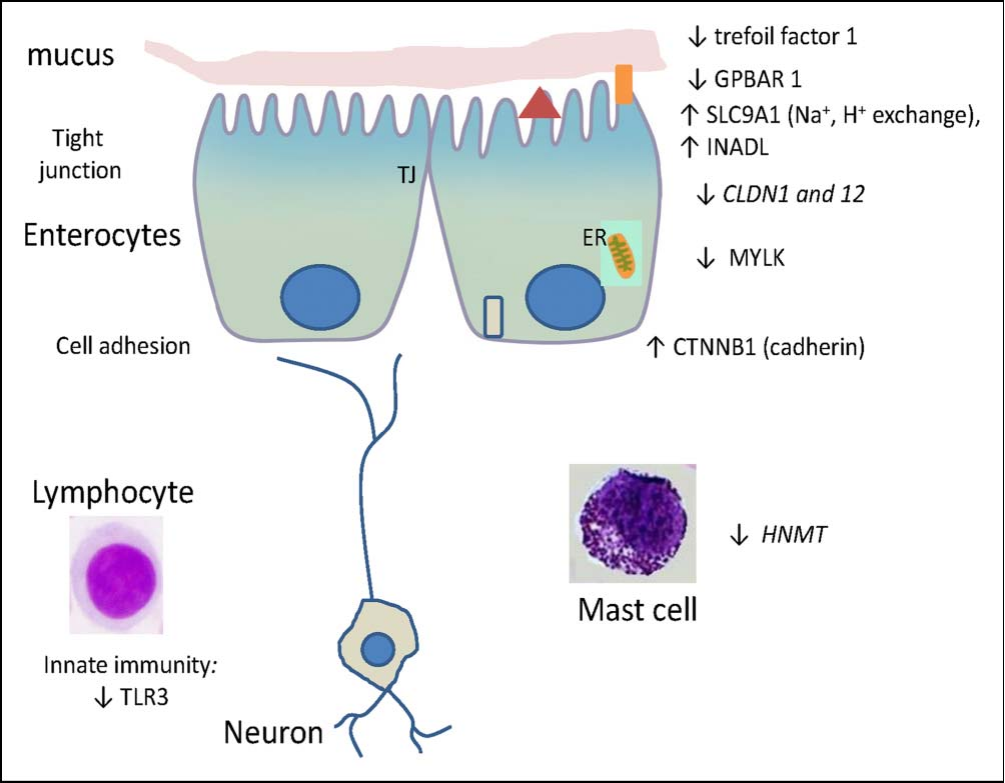
Messenger RNA expression in ileal mucosal biopsies from patients with constipation-predominant irritable bowel syndrome compared with healthy controls. Note the reduced expression of genes controlling G-protein–coupled bile acid receptor 1 (GPBAR1), myosin light chain kinase (MYLK in barrier function), epithelial repair (trefoil factor 1), and innate immunity (toll-like receptor 3, TLR3). By contrast, the ileal mucosal biopsies from patients with constipation-predominant irritable bowel syndrome compared with healthy controls had increased cadherin cell adhesion mechanisms (catenin beta 1, CTNNB1) and in transport genes, solute carrier family 9 member A1 (SLC9A1 [Na-H exchanger]) and inactivation-no-afterpotential D-like (INADL [indirect effect on ion transport]). HNMT, histamine n-methyl transferase.

**Figure 2.**
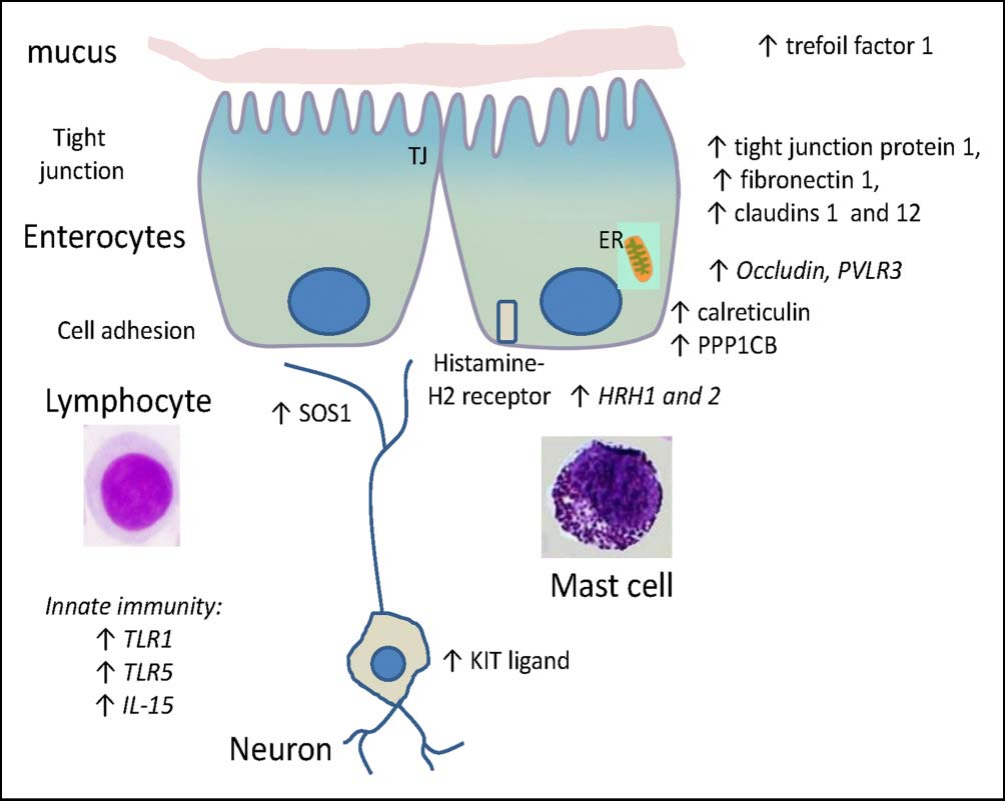
Messenger RNA expression in ileal mucosal biopsies from patients with diarrhea-predominant irritable bowel syndrome compared with patients with constipation-predominant irritable bowel syndrome. Note the upregulation of several genes controlling barrier proteins (tight junction protein 1, fibronectin 1, and claudins 1 and 12), repair function (trefoil factor 1) and cellular functions such as genes controlling cell development (KIT ligand), protein phosphatase (protein phosphatase 1 catalytic subunit beta, PPP1CB), guanine nucleotide exchange (Sons of Sevenless [*Drosophila*] homolog 1, SOS1), and function of endoplasmic reticulum (calreticulin), potentially reflecting protein synthesis. There were also numerical increases (*P* < 0.10) in expression of genes for histamine H-1 and H-2 receptors (HRH1 and 2) and markers of innate immunity or inflammation (toll-like receptor 1 and 5 [TLR1 and 5] and interleukin-15 [IL-15]). ER, endoplasmic reticulum; TJ, tight junction.

In Figures 1 and 2, the statistically significant differences in mRNA expression are shown; in addition, expression data that showed nominal values of 0.05 > *P* < 0.10 are shown in italics in Figures 1 and 2. The final column in Table 2 and the directions of the arrows in Figures 1 and 2 show the relative regulation of the genes in the 2 sets of comparisons of interest, i.e., IBS-C vs health, and IBS-D vs IBS-C. Fold change and fold regulation of mRNA according to group in ileal biopsies of IBS-C vs health and IBS-D vs IBS-C are shown in Table 3.

**Table 3.** Fold change and fold regulation of mRNA according to group in ileal biopsies IBS-C vs HC and IBS-D vs IBS-C

Symbol	Average fold change IBS-C/HC	Average fold regulation IBS-C/HC	Average fold change IBS-D/IBS-C	Average fold regulation IBS-D/IBS-C
Cell adhesion, tight junctions, or barrier function				
TFF1	0.580	−1.724	2.109	2.109
FN1	0.716	−1.397	1.734	1.734
CLDN1	0.610	−1.640	2.150	2.150
CLDN12	0.765	−1.307	1.415	1.415
PVRL3 (Nectin3)	0.796	−1.256	1.331	1.331
OCLN	0.878	−1.139	1.328	1.328
MYLK	0.767	−1.305	1.365	1.365
TJP1	0.896	−1.117	1.425	1.425
FGFR4	1.062	1.062	1.367	1.367
Neurotransmitters, exchangers, and receptors				
GPBAR1	0.674	−1.483	1.285	1.285
HNMT	0.685	−1.461	1.423	1.423
HRH1	0.932	−1.073	1.217	1.217
HRH2	0.644	−1.554	2.260	2.260
SLC9A1	1.300	1.300	1.024	1.024
KITLG	0.754	−1.327	1.672	1.672
INADL (PATJ)	1.262	1.262	0.868	−1.152
SOS1	0.914	−1.094	1.256	1.256
Cell kinetics				
CTNNB1	1.253	1.253	0.865	−1.156
CALR	0.821	−1.218	1.556	1.556
PPP1CB	0.910	−1.099	1.166	1.166
Innate immunity and inflammation				
TLR1	0.636	−1.573	1.952	1.952
TLR3	0.720	−1.389	1.182	1.182
TLR5	0.768	−1.302	1.486	1.486
IL15	0.807	−1.239	1.279	1.279

Average fold regulation was calculated by the formula: −(1/fold change), if fold change <1. When the fold change is >1, the fold change and fold regulation are equal.

CALR, calreticulin; CLDN1, claudin-1; CLDN12, claudin-12; CTNNB1, catenin beta 1; FGFR4, fibroblast growth factor receptor 4; FN1, fibronectin 1; GPBAR1, G-protein–coupled bile acid receptor 1; HC, healthy controls; HNMT, histamine n-methyl transferase; HRH1, histamine receptor H1; HRH2, histamine receptor H2; IBS-C, constipation-predominant irritable bowel syndrome; IBS-D, diarrhea-predominant irritable bowel syndrome; IL15, interleukin 15; INADL, inactivation-no-afterpotential D-like (also known as PATJ); KITLG, KIT ligand; mRNA, messenger RNA; MYLK, myosin light chain kinase; OCLN, occludin; PPP1CB, protein phosphatase 1 catalytic subunit beta; PVRL3, poliovirus-receptor-related 3 (also known as nectin 3); SLC9A1, solute carrier family 9 member A1; SOS1, Sons of Sevenless (*Drosophila*) homolog 1; TFF1, trefoil factor 1;TJP1, tight junction protein 1; TLR1, toll-like receptor 1; TLR3, toll-like receptor 3; TLR5, toll-like receptor 5.

Ileal mucosal biopsies from patients with IBS-C relative to healthy controls (Figure 1) showed reduced expression of genes controlling GPBAR1 receptor, myosin light chain kinase (MYLK in barrier function), epithelial repair (TFF1), and innate immunity (TLR3). By contrast, the ileal mucosal biopsies from IBS-C compared with healthy controls showed increased cadherin cell adhesion mechanisms (CTNNB1) and in transport genes SLC9A1 (Na-H exchanger) and INADL (indirect effect on ion transport). There were also borderline reductions in mRNA for 2 barrier proteins (CLDN1 and 12) and for the metabolism of histamine n-methyl transferase (HNMT).

Prominent and significant differences in ileal mRNA expression in patients with IBS-D relative to IBS-C (Figure 2) were upregulation of several genes controlling barrier proteins (TJP1, FN1, and CLDN1 and 12), repair function (TFF1) and cellular functions such as genes controlling cell development (KITLG), protein phosphatase (PPP1CB), guanine nucleotide exchange (Sons of Sevenless [*Drosophila*] homolog [SOS1]), and function of endoplasmic reticulum (CALR), potentially reflecting protein synthesis. There were also numerical increases (*P* < 0.10) in the expressions of genes for histamine H-1 and H-2 receptors and markers of innate immunity or inflammation (TLR1 and 5 and IL-15).

### Protein measurements by Western blot

**Figure 3.**
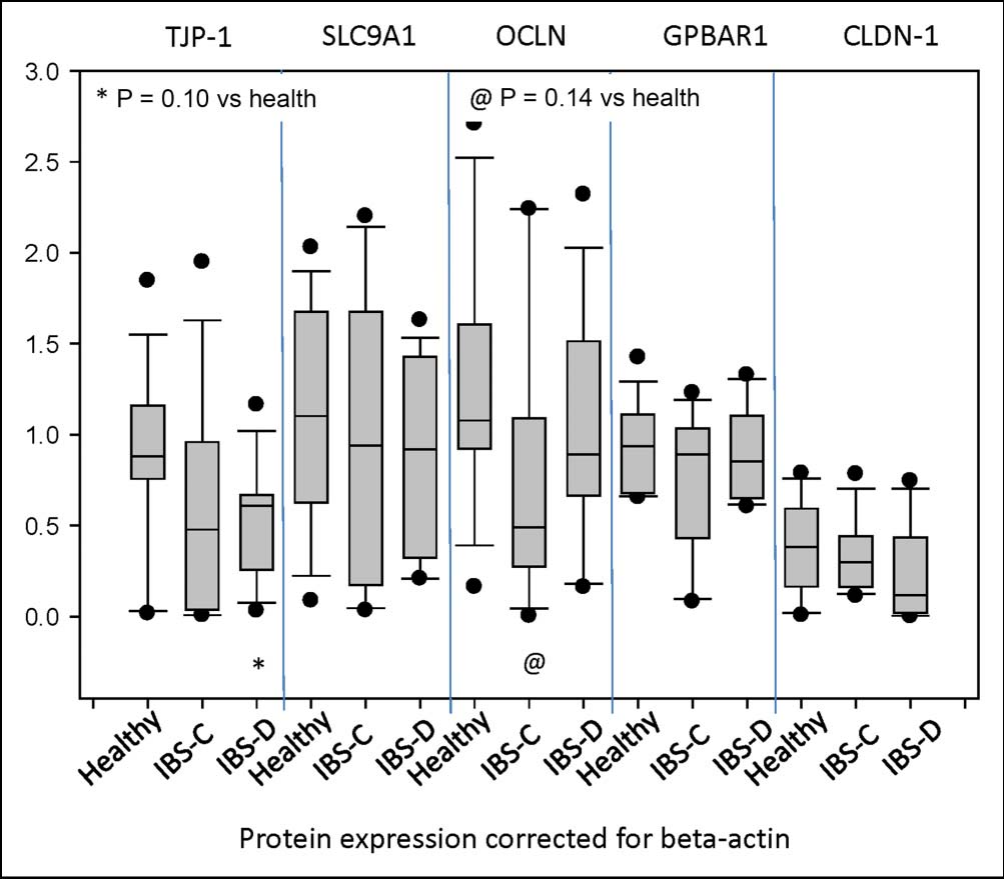
Expression of barrier proteins (tight junction protein 1 [TJP-1], occludin [OCLN], and claudin-1 [CLDN-1]), sodium hydrogen exchanger (solute carrier family 9 member A1 [SLC9A1]), and the bile acid transporter (G-protein–coupled bile acid receptor 1 [GPBAR1]) in ileal biopsies from healthy controls and from patients with constipation-predominant irritable bowel syndrome (IBS-C) or diarrhea-predominant irritable bowel syndrome (IBS-D). These measurements are based on Western blot quantification and 3 group comparisons based on ANOVA on ranks. Note the numerically lower expression of TJP-1 in biopsies from patients with IBS-D compared with healthy controls.

A descriptive comparisons of the 5 proteins tested between the healthy, IBS-C, and IBS-D groups showed no overall significant differences in the protein expression levels for TJP1, occludin, claudin 1, SLC9A1, or GPBAR1 (assessed by ANOVA on ranks). However, there were nonsignificant reductions in the ileal biopsies for TJP1 in IBS-D and for occludin in IBS-C compared with healthy controls (Figure 3). shows the ratios of each protein relative to the mean Table 4level in normal control mucosa run in the same gel. The ratio data relative to healthy controls suggest reduced protein levels (95% confidence interval does not cross the value of 1) for TJP1 and SLC9A1 in IBS-C and IBS-D and for occludin in IBS-C. There were no differences in the protein expressions of SLC9A1 or GPBAR1 between the 3 groups.

**Table 4.** Comparison of 5 proteins in ileal mucosa of patients with IBS-C or IBS-D expressed as ratios relative to biopsies from healthy controls estimated within Western blots

Protein	Mean	Median	95% CI of median
IBS-C group			
TJP-1	0.724	0.515	0.327–0.795
SLC9A1	1.054	0.773	0.179–0.974
OCLN	0.768	0.467	0.199–0.850
GPBAR1	0.762	0.834	0.579–1.062
CLDN	1.302	1.166	0.281–2.402
IBS-D group			
TJP-1	0.619	0.471	0.280–0.949
SLC9A1	0.829	0.807	0.654–0.903
OCLN	1.007	0.800	0.517–1.361
GPBAR1	0.980	0.870	0.664–1.237
CLDN	0.700	0.242	0.0395–1.626

Note the reduced expression shown by ratios that do not overlap 1.0 in the 95% CIs for TJP-1, SLC9A1 (for both IBS-C and IBS-D) and for OCLN in IBS-C.

CI, confidence interval; CLDN, claudin; GPBAR1, G-protein–coupled bile acid receptor 1; IBS-C, constipation-predominant irritable bowel syndrome; IBS-D, diarrhea-predominant irritable bowel syndrome; OCLN, occluding; SLC9A1, solute carrier family 9 member A1; TJP1, tight junction protein 1.

### Clustering analysis of pathways and mechanisms

**Figure 4.**
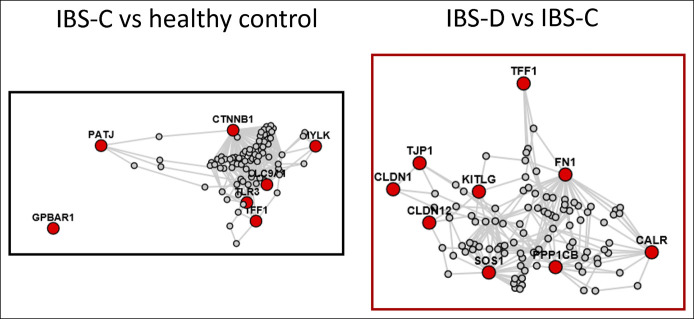
Lens for enrichment and network studies analysis of altered expression of messenger RNA (mRNA) in ileal biopsies of healthy controls and patients with constipation-predominant irritable bowel syndrome (IBS-C) and diarrhea-predominant irritable bowel syndrome (IBS-D). Note in the left panel the connectivity of mechanisms involved in ion absorption or secretion (particularly, solute carrier family 9 member A1 and PATJ [also known as inactivation-no-afterpotential D-like) as well as the unconnected downregulated G-protein–coupled bile acid receptor 1 (GPBAR1) in IBS-C. The right panel shows connectivity for the increased mRNA expression related to barrier function, as well as cell development, kinetics, and repair. CALR, calreticulin; CLDN, claudin; CTNNB1, catenin beta 1; FN1, fibronectin 1; KITLG, KIT ligand; MYLK, myosin light chain kinase; PPP1CB, protein phosphatase 1 catalytic subunit beta; SOS1, Sons of Sevenless (*Drosophila*) homolog 1; TFF1, trefoil factor 1; TJP1, tight junction protein 1; TLR, toll-like receptor.

Having identified the nominally, univariately significant (*P* < 0.05) upregulation or downregulation of genes in the ileal mucosa, we applied lens for enrichment and network studies analysis and plotted connectivity among the genes associated with barrier, ion channels, receptors, and immune activation (Figure 4).

In the analysis of mRNA in patients with IBS-C compared with controls (Figure 3, left panel), the connectivity of mechanisms involved in ion absorption or secretion (particularly, SLC9A1 and INADL), as well as the unconnected downregulated GPBAR1 receptor, was noted.

The molecules of greatest interest in the network diagram for IBS-D relative to IBS-C (Figure 4, right panel) are linked by intermediate molecules, and they illustrate connectivity for the widely increased mRNA expression related to barrier function, as well as cell development, kinetics, and repair.

### Gene set enrichment analysis

The pathways involved in the actions of the altered mRNA expression are detailed in Figure 4 and include important roles of cellular adhesion and junctions in IBS-D and of responses to abiotic stimuli in patients with IBS-C.

**Figure 5.**
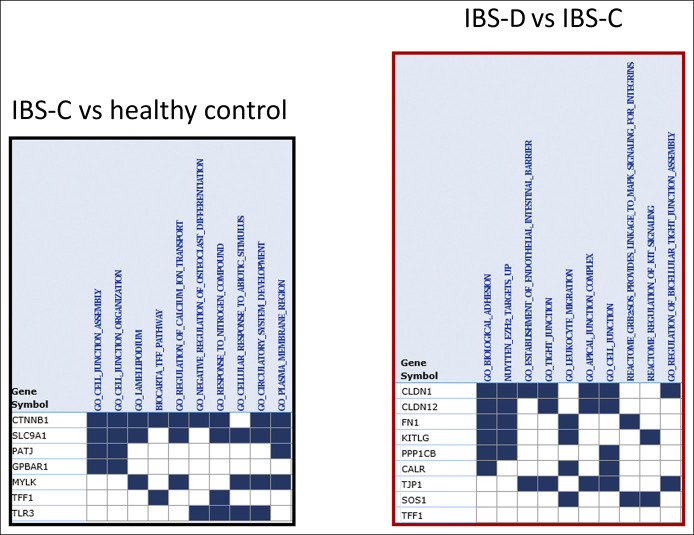
Gene set enrichment analysis of altered expression of messenger RNA (mRNA) in ileal biopsies of healthy controls and patients with constipation-predominant irritable bowel syndrome (IBS-C) and diarrhea-predominant irritable bowel syndrome (IBS-D). Left panel: Note, in patients with IBS-C compared with healthy controls, the involvement of genes associated with cellular junction assembly and organization, and regulation of fluid/electrolyte exchange. Right panel: In biopsies from patients with diarrhea-predominant irritable bowel syndrome (IBS-D) compared with IBS-C, note the genes related to altered tight junctions, apical complexes, cellular adhesion, leucocyte migration, and cell development. CALR, calreticulin; CLDN, claudin; CTNNB1, catenin beta 1; FN1, fibronectin 1; GBPAR1, G-protein–coupled bile acid receptor 1; KITLG, KIT ligand; MYLK, myosin light chain kinase; PATJ (also known as inactivation-no-afterpotential D-like); PPP1CB, protein phosphatase 1 catalytic subunit beta; SLC9A1, solute carrier family 9 member A1; SOS1, Sons of Sevenless (*Drosophila*) homolog 1; TFF1, trefoil factor 1; TJP1, tight junction protein 1; TLR3, toll-like receptor 3.

For the gene set with altered fold regulation in IBS-C compared with healthy controls, the GSEA analysis identified the overlap matrix (top 10) among the 7 genes of interest (Figure 5a) including cellular junction assembly and organization and regulation of fluid/electrolyte exchange or responses to abiotic stimuli at the enterocyte levels. All were associated with an FDR Q-value <0.02, with cell junction assembly or organization at *P* ≤ 10^−4^.

For the 9 genes with significantly different fold regulation in IBS-D compared with IBS-C, the GSEA analysis identified the top 10 overlap genes (Figure 5b) which were dominated by altered tight junctions, apical complexes, cellular adhesion, leucocyte migration, and cell development (KIT signaling). All were associated with FDR *Q* values <10^−5^.

## DISCUSSION

Our study shows that, in ileal mucosal biopsies, mRNA expression of several genes were significantly upregulated or downregulated in patients with IBS-D or IBS-C.

### Findings in patients with IBS-D

mRNA expression of several genes were significantly upregulated in IBS-D relative to IBS-C including trefoil factor 1, genes controlling several tight junction proteins, and guanine nucleotide exchange (SOS1), and there were borderline increases in toll-like receptor genes and histamine H-1 and H-2 receptors. The functional consequences anticipated from these gene changes are:

1. Decreased mucosal permeability as a result of the increased expression of mRNA for tight junction protein 1, fibronectin 1, claudins 1 and 12, and borderline increases in occludin and nectin 3. Decreased ileal mucosal permeability could decrease passive permeation of fluid and electrolytes from the plasma to the lumen, e.g., in response to arrival of osmotically active digestive products in the ileum such as lactose in patients with hypolactasia, or fructans ingested in the diet, given the inability of the human small intestine to digest or absorb fructans. By contrast, there was no increased expression of SLC9A1, which would result in greater fluid absorption through the NHE3 channel. These data in IBS-D suggest the hypothesis that barrier genes in ileal mucosa increase to compensate for secretory mechanisms that are activated (e.g., 5-HT-related, as detailed in the Introduction) or the increased expression of the increased mucosal expression of SOS1 (discussed below).
2. Increased production of histamine receptors may conceivably result in ileal secretion. Administration of histamine in dogs was associated with extensive capillary leak and intestinal secretion of bicarbonate-rich fluid (30), and increased capillary and transmucosal permeability (31). Histamine also activated immune mechanisms or inflammation, or mast cell activation in patients with IBS-D (32). In the patients with IBS-D (not selected for abnormal bile acid kinetics), we did not observe significant changes in *SLC10A2* or *NR1H4*, which are the genes for ASBT and farnesoid X receptor, respectively, and are associated with bile acid diarrhea.

In patients with bile acid diarrhea, Johnston et al. observed low ileal FGF19 mRNA, which was associated with worse bile acid loss (23).

Despite the evidence of activation of immune mechanisms or inflammation in patients with IBS-D (33), we did not observe statistically significant alterations in the expression of immune or inflammatory mediators. By contrast, the mRNA expressions of TLR1 and 5 (both of which recognize pathogenic bacteria) and IL15 (which is involved in maintaining naive, effector and memory T cells, and in reactivation of memory T cells) were borderline (0.05 > *P* < 0.10) increased in IBS-D compared with IBS-C. It is conceivable that there are different subgroups of immune modulation within the IBS-D phenotype. In our previous study of markers of immune activation or inflammation in colonic mucosa from patients with IBS-D, we also observed increased mRNA expression of C4BPA, decreased CCL20, and no change in IF1T3 or TNFSF15 (17); however, none of these cytokines showed altered expression in the current studies of ileal biopsies.

The increased KIT ligand gene in IBS-D may suggest increased cellular development, but the effect on motility or bowel function would be uncertain. SOS1 expression was also increased in patients with IBS-D compared with IBS-C. SOS1 is a guanine nucleotide exchange factor. A guanine nucleotide exchange factor, Epac (exchange protein directly activated by cAMP), seems to be involved in intestinal secretion (34–36). Therefore, the increased mucosal expression of SOS1 may be a factor involved in intestinal secretion, for which the increased barrier function may serve as an adaptive, compensatory response.

### Findings in patients with IBS-C

Ileal mucosal biopsies from patients with IBS-C relative to healthy controls showed reduced mRNA expression of GPBAR1 receptor, markers of cell repair, and innate immunity (TLR3), and increased expression of SLC9A1 (Na-H exchanger) and INADL (indirect effect on ion transport). Overall, these data are consistent with the hypothesis that the mucosa absorbs more sodium and water, and it would be expected that there would be reduced responses to endogenous bile acids in the ileum, since the expression of GPBAR1 is reduced. There were borderline changes in some of the tight junction proteins, but not in others.

The changes in mRNA expression may conceivably be associated with increased fluid or electrolyte secretion, such as the upregulation of *InaD-like* (*Drosophila*) gene for which there is a human analog *INADL* gene producing a protein (33) that encodes multiple PDZ domain proteins that may enhance ion transport, and a related tight junction-associated multi-PDZ protein PATJ (PALS1-associated TJ protein) that connects and stabilizes apical and lateral components of tight junctions in human intestinal cells (37). Studies in knock-out mice show that GPBAR1 mediates bile acid effect on colonic transit, and the deficiency of such leads to decreased defecation frequency and lower stool water content (38).

The borderline decreased gene expression of HNMT in IBS-C relative to healthy controls may result in reduced histamine metabolism. Histamine originates from enteroendocrine cells or mast cells. The density of mast cells is increased in the jejunal mucosa of nonatopic patients with IBS-D (39), but there are limited reports of mast cell density in IBS-C. These reports consistently show increased mast cell density in both IBS-C and IBS-D, with 1 study suggesting correlation of mast cell density to pain perception (40). However, no studies, thus far, have studied the function of these mast cells, and the increased numbers may be a compensatory change to reduced function as documented in the current study which showed reduced HNMT expression in the mucosal biopsies (41–43). Decreased HNMT would be expected to result in increased ileal fluid secretion (perhaps as an adaptive response to the constipation) and possibly contribute to pain in IBS-C in view of the demonstrated *in vitro* effects of histamine on nociceptors (32). In addition, the decreased expression for the enzyme metabolizing histamine is consistent with increased release or supernatant content of histamine from colorectal mucosal biopsies from patients with IBS (44–48). Overall, the reduced expression of histamine metabolizing enzyme may also serve as an adaptive response to the constipation in patients with IBS-C.

The measurements of selected proteins in mucosal biopsies from patients with IBS-C showed reduction in 1 barrier (occludin) which is generally consistent with reduced mRNA expression of some barrier-related genes. However, other information seems to be contradictory because SLC9A1 mRNA expression was increased and the protein product NHE1 was decreased in ileal biopsies from patients with IBS-C.

### Relationship of current observations to previous studies and importance of studies of distal small intestine

Our observations on ileal mucosal biopsies in patients with IBS-D are consistent with some of our previous observations (20) on mRNA in duodenal mucosal biopsies from 15 patients with IBS-D, such as upregulation of immune functions (TLR3, IL15, and MAPKAPK5), as well as the upregulation of genes related to barrier functions (TJP1, 2, and 3 and CLDN). However, in the ileal mucosa, we did not observe alterations in other genes related to immune functions (IL-1β, TGF-β1, and CCL20) or antigen detection (TLR1 and 8).

Given the important role of the small intestine in water regulation of the intraluminal content and the role of the terminal ileum in bile acid homeostasis that can lead to bile acid diarrhea (48), we believe the observations on distal ileal mucosal mRNA expression provide important insights on the pathogenesis of IBS. These studies complement clinical observations in IBS. For example, Li et al. showed increased small bowel permeability with no change in colonic permeability in patients with IBS-D compared with controls; the degree of permeability increase correlated with psychological effects and quality of life (49). In the same study, evaluation of terminal ileum mRNA expression in the patients with IBS-D with increased permeability compared with those without increased permeability identified 185 genes differentially expressed in the patients with IBS-D. Those studies showed that increased mucosal permeability was associated with gene clusters in functions such as immune system processing, membrane adhesion, and protein/receptor binding, among others. Among the junction proteins, mRNA expression of ZO-1,2,3 and occludin were not significantly different, and claudin-4 was significantly higher in patients with increased small intestinal permeability compared with those without increased permeability (49). Li et al. also found upregulation of 1 inflammatory cytokine (CXCL5) with decreased expression of CXCL13 and CXCR4, suggestive of variable inflammation-related mechanisms with immune system genes being differentially expressed in patients with increased intestinal permeability.

Biopsies from duodenum, jejunum, and ileum in 20 patients with IBS-D, 18 patients with IBS-C, and 20 healthy controls showed no differences in enterochromaffin cell distribution, as well as lower levels of enterochromaffin (5-HT) throughout the small intestine in patients with IBS-C compared with healthy controls, and increased mast cell numbers in patients with IBS-C and IBS-D in the terminal ileum compared with healthy controls (41).

### Unanswered questions and future research

Our study is limited by a relatively small sample size and the use of a limited number of gene measurements rather than an unrestricted evaluation using RNA sequencing. Our eventual plan is to conduct RNA sequencing and proteomics analysis on biopsies from the terminal ileum and right and left colon in a larger number of patients with the same 3 conditions (60 patients with IBS-D, 30 with IBS-C, and 30 healthy controls). However, our current study provides important insights on alterations in gene expression in ileal mucosa that are biologically plausible and sets the stage for the hypothesis-testing study mentioned above. Another limitation is the lack of concordance in the data from mRNA expression and protein measurements, e.g., the increased SLC9A1 mRNA, but reduced protein in IBS-C. A final limitation of the current study is the use of nominal *P* values which were uncorrected for false detection rate.

In summary, this analysis of gene expression studies in terminal ileal biopsies shows alterations that would be associated with plausible biological changes in patients with IBS-D relative to IBS-C and between IBS-C and healthy controls. The observations in the current study provide a step to further understand potential etiopathogenetic mediators, as well as biological pathways involved in IBS.

## CONFLICTS OF INTEREST

**Guarantor of the article:** Michael Camilleri, MD.

**Specific author contributions:** X.J.W.: fellow, patient recruitment, biopsy acquisition, and coauthorship of manuscript. P.C.: expression analysis and coauthorship of manuscript. V.C. and D.B.M.: patient recruitment, biopsy acquisition, and coauthorship of manuscript. A.L.T. and S.M.: study coordinator. M.C.: principal investigator, conceptualization, analysis and interpretation, and authorship of manuscript.

**Financial support:** This study was supported by NIH grant R01-DK115950 (M. Camilleri). The study was made possible by CCaTS grant UL1-TR000135 from the National Center for Advancing Translational Sciences (NCATS), a component of National Institutes of Health (NIH). Its contents are solely the responsibility of the authors and do not necessarily represent the official view of NIH.

**Potential competing interests:** None to report.

**Trial registry:** These studies were performed as part of an NIH-funded grant R01-DK115950 (Federal Award Date: December 27, 2017). The grant trial was registered in ClinicalTrials.gov: #NCT02163213.

**Study Highlights**

**WHAT IS KNOWN**

✓ Gene and protein expressions in colonic and upper small intestinal mucosa of patients with irritable bowel syndrome (IBS) have been described previously; these studies focused mainly on patients with IBS-diarrhea.

**WHAT IS NEW HERE**

✓ Completely novel observations of gene and protein expressions in ileal mucosa from patients with both IBS-diarrhea and IBS-constipation are provided and illustrate a role of ileal mucosal dysfunction in IBS including barrier dysfunction in IBS-diarrhea and alterations in absorption/secretion mechanisms in IBS-constipation.

**TRANSLATIONAL IMPACT**

✓ Novel therapeutic directions that restore normal barrier function and ion transport may correct the pathobiologies identified in IBS subgroups and may impact the clinical manifestations of IBS.

## Supplementary Material

SUPPLEMENTARY MATERIAL
