# Supplementary material for: Differential mRNA Expression in Ileal Mucosal Biopsies of Patients With Diarrhea- or Constipation-Predominant Irritable Bowel Syndrome
Source: Clin Transl Gastroenterol. 2021 Apr 12;12(4):e00329. doi: 10.14309/ctg.0000000000000329 (PMC8043738; doi:10.14309/ctg.0000000000000329)
Supplement: SUPPLEMENTARY MATERIAL [file ct9-12-e00329-s001.docx]

**SUPPLEMENTAL MATERIALS**

**Acquisition and Storage of Biospecimens**

The mucosal biopsies from patients with IBS-D or IBS-C and healthy controls were acquired at the time of colonoscopy performed explicitly for this research study. Patients received conscious sedation consisting exclusively of midazolam (Versed®, a benzodiazepine) and sublimaze (Fentanyl®, a mu-opioid receptor agonist).

Biopsies were preserved in a solution of RNAlater and stored at -80°C until the time of assay of gene expression or Western blot assays for selected proteins.

**mRNA Extraction**

For mRNA expression, RNA was purified from terminal ileal biopsies using the Qiagen RNAeasy Kit, including on-column DNAse treatment to remove genomic DNA. RNA quality was assessed on the Agilent Bioanalyzer. The resulting RNA (RIN >7) was reverse transcribed using the RT^2^ First Strand Kit (Qiagen), and samples were analyzed for expression by a Custom Profiler RT^2^ PCR Array (Qiagen).

**Gene Expression Method by RT^2^ PCR Array**

The RT^2^-Profiler-PCR-Array data examined 78 genes on 42 samples (11 IBS-D, 17 IBS‑C patients, and 14 healthy controls). In addition to the 78 genes, there were three housekeeping genes (B2M, ACTB, and GAPDH), and another three (HGDC, RTC, PPC) were assay controls. Therefore, the custom array analysis focused on 78 genes whose general functions are included in Table 1 in the main manuscript. Since SYBR green intercalates double stranded DNA, post-polymerase chain reaction (PCR) melting curve analysis was run. Samples without single specific products were removed from the statistical analysis. Due to significant variation in B2M expression across biopsies, we used the mean of GAPDH and ACTB expression as housekeeping gene in the assessment of differential expression of each gene corrected for expression of the control genes. To control for DNA contamination introduced during reaction setup, a no template control reaction replacing template with water, as well as a no reverse transcription reaction were run with the assays.

**Protein Measurements by Western Blots**

Whole cell lysates were isolated from human ileal mucosal biopsies with the RIPA Lysis Buffer System (Santa Cruz Biotechnology). Briefly, tissue samples were homogenized followed by sonication in RIPA lysis buffer containing protease inhibitors. The homogenate was centrifuged at 10,000 x g. The supernatant was centrifuged again at 10,000 x g, and the resulting supernatant was used for the Western blot analysis. Protein concentrations were determined by Pierce™ BCA Protein Assay (Thermo Scientific) and read on the NanoDrop™ (Thermo Scientific). A total of 20 µg proteins were separated by use of 4-15% Mini-PROTEAN® TGX™ gels (Bio-Rad) and wet transferred onto 0.2 µm nitrocellulose membranes (Bio-Rad). The membranes were blocked with 5% milk in TBS/0.1% Tween and washed, and primary antibodies diluted in 5% BSA TBST were applied overnight at 4°C. β-actin was used for normalization of protein loading for all the proteins of interest. Membranes were washed with TBST and incubated with horseradish peroxidase-conjugated secondary antibody (anti-rabbit 7074, Cell Signaling, or anti-mouse sc2962, Santa Cruz Biotechnology), and were then visualized with chemiluminescence SuperSignal™ West Pico Plus (Thermo Scientific) and autoradiography. Band densities were quantified with the Image J 1.52p (National Institutes of Health, USA). In summary, we measured the proteins ZO-1(TJP1) (Invitrogen 61-7300, 1:250), occludin (Cell Signaling 91131(E6B4R),1:1000) claudin-1(Invitrogen 37-4900, 1:200), GPBAR1 (Abcam ab72608, 1:1000) and SLC9A1 (NHE1) (Abcam ab67314, 1:500) and the housekeeper β-actin (, Sigma Aldrich A2066, 1:5000).

**Pathway and Cluster Analyses**

We used a publicly available informatics tool to appraise the potential pathways or clustered mechanisms that are altered in the duodenal mucosa of patients with IBS-D, relative to controls. The Lens for Enrichment and Network Studies (LENS) of proteins provides interactome analysis of the list of human genes examined in this study in order to explore the network of protein-protein interactions that connects them (1). LENS computes associations of the genes in the interactome to pathways, and assesses statistics of network connectivity of these genes compared against connectivity of randomly selected genes. Sources of data for the network generation are direct (biophysical) interactions from HPRD and BioGRID, and pathways are obtained from Reactome (<http://severus.dbmi.pitt.edu/LENS/index.php>).

**Analysis of Gene Expression and Overlap of Pathways Using Gene Set Enrichment Analysis**

Gene Set Enrichment Analysis (GSEA) is a computational method that determines whether an *a priori* defined set of genes shows statistically significant, concordant differences between two biological states. GSEA has been applied widely as a tool for gene set analyses.

For pathway investigation, we included the gene set sub-catalogs C1, C2 and C5 from [MolecularSignatures Database (MSigDB) v5.1,](http://software.broadinstitute.org/gsea/msigdb/index.jsp) a collection of annotated gene sets for use with GSEA software (<http://www.broad.mit.edu/gsea>). The C1 catalog includes gene sets corresponding to human chromosomes and cytogenetic bands. The C2 catalog includes gene sets that are involved in specific metabolic and signaling pathways collected from various sources such as online pathway databases, publications in PubMed, and knowledge of domain experts. The C5 catalog pertains to biological processes, cellular components and molecular functions (2‑4). These pathway investigations were conducted separately based on the genes that were significantly over- or under-expressed in the two comparisons of interest (healthy vs. IBS-C, and IBS-D vs. IBS-C).

**References**

1. Handen A, Ganapathiraju MK. LENS: web-based lens for enrichment and network studies of human proteins. BMC Med Genomics 2015;8:S2.

2. Dinu I, Potter JD, Mueller T, et al. Gene-set analysis and reduction. Brief Bioinform 2009;10:24-34.

3. Dinu I, Potter JD, Mueller T, et al. Improving gene set analysis of microarray data by SAM-GS. BMC Bioinformatics 2007;8:242.

4. Liu Q, Dinu I, Adewale AJ, et al. Comparative evaluation of gene-set analysis methods. BMC Bioinformatics 2007;8:431.

**Supplemental Table 1. Genes of interest included in the RT-PCR analysis of terminal ileal mucosa**

| **Gene Symbol** | **Refseq #** | **Official Full Name** | **Gene Function** |
| --- | --- | --- | --- |
| SLC6A4 | NM_001045 | 5-HT transporter | 5-HT transporter |
| QPRT | NM_014298 | Quinolinic acid phosphoribosyltransferase | 5-HT metabolism |
| TDO2 | NM_005651 | Tryptophan 2,3-dioxygenase |  |
| TPH1 | [NM_004179](http://www.ncbi.nlm.nih.gov/entrez/query.fcgi?CMD=search&DB=gene&term=NM_004179&doptcmdl=Graphics) | Tryptophan hydroxylase 1 |  |
| AADAT | NM_182662 | Kynurenine aminotransferase 2 (alias KAT2) | 5-HT/tryptophan metabolism |
| CCBL2 | NM_001008661 | Kynurenine aminotransferase 3 (alias KAT3) |  |
| GOT2 | NM_002080 | Kynurenine aminotransferase 4 (alias KAT4) |  |
| KMO | NM_003679 | Kynurenine 3-monooxygenase |  |
| KYNU | NM_003937 | Kyureninase |  |
| IDO1 | NM_002164 | Indoleamine 2,3-dioxygenase | Breakdown of non-dietary tryptophan |
| IDO2 | NM_194294 | indoleamine 2,3-dioxygenase 2 |  |
| NR1H4 | NM_005123 | Nuclear receptor subfamily 1, group H, member 4 | FXR receptor |
| SLC10A2 | NM_000452 | Solute carrier family 10 member 2 cotransporter | ASBT (bile acid) |
| TFF1 | NM_003225 | Trefoil factor 1 | Mucin synthesis |
| CLDN1 | NM_021101 | Claudin 1 | Barrier |
| FN1 | NM_002026 | Fibronectin 1 |  |
| OCLN | NM_002538 | Occludin |  |
| TJP1 | NM_175610 | Tight junction protein 1 (zona occludens 1) |  |
| MYLK | NM_053025 | Myosin Light Chain Kinase |  |
| CLDN2 | NM_020384 | Claudin 2 |  |
| CLDN3 | NM_001306 | Claudin 3 |  |
| CLDN4 | NM_001305 | Claudin 4 |  |
| CLDN7 | NM_001307 | Claudin 7 |  |
| CLDN12 | NM_012129 | Claudin 12 |  |
| CLDN15 | NM_014343 | Claudin 15 |  |
| TJP2 | NM_004817 | zona occludens 2 |  |
| TJP3 | NM_014428 | zona occludens 3 |  |
| CTNNA1 | NM_001903 | Catenin (cadherin-associated protein), alpha 1 | Cell adhesion |
| CTNNB1 | NM_001904 | Catenin (cadherin-associated protein), beta1 |  |
| PVRL3 | NM_015480 | Poliovirus signaling-related 3 (aka nectin-3) |  |
| KITLG | NM_003994 | Kit-ligand, Stem cell factor | Cell development |
| DLG1 | NM_004087 | Discs, large homolog 1 (Drosophila) | Cell kinetics |
| MAPKAPK5 | NM_003668 | Mitogen-activated protein kinase-activated protein kinase 5 |  |
| MKNK2 | NM_017572 | MAP kinase interacting serine/threonine kinase 2 |  |
| MAGI1 | NM_004742 | Membrane associated guanylate kinase, WW and PDZ domain containing 1 | Cell metabolism |
| MPP5 | NM_022474 | Membrane protein, palmitoylated 5 (MAGUK p55 subfamily member 5) |  |
| MPP7 | NM_173496 | Membrane protein, palmitoylated 7 (MAGUK p55 subfamily member 7) |  |
| PPP1CB | NM_002709 | Protein phosphatase 1, catalytic subunit, beta isozyme |  |
| PPP2R5C | NM_001161725 | Protein phosphatase 2, regulatory subunit Beta |  |
| FGFR4 | NM_002011 | Fibroblast growth factor receptor 4 | Cell proliferation |
| CALR | NM_004343 | CALReticulin | Endoplasmic reticulum function |
| SOS1 | [NM_005633](http://www.ncbi.nlm.nih.gov/entrez/query.fcgi?CMD=search&DB=gene&term=NM_005633&doptcmdl=Graphics) | Son of sevenless homolog 1 (Drosophila) | Guanine nucleotide exchange |
| HNMT | NM_006895 | Histamine n-methyltransferase | Mast cell |
| HRH1 | NM_000861 | Histamine receptor 1 | Histamine receptor |
| HRH2 | NM_022304 | Histamine receptor 2 |  |
| C4BPA | NM_000715 | Complement component 4 binding protein, alpha | Inflammation |
| CCL20 | NM_004591 | Chemokine (C-C motif) ligand 20 |  |
| IFIT3 | NM_001549 | Interferon-induced protein with tetratricopeptide repeats 3 |  |
| TNFSF15 | NM_005118 | Tumor necrosis factor (ligand) superfamily, member 15 |  |
| IFNG | NM_000619 | Interferon-gamma |  |
| IL1B | NM_000576 | Interleukin-1beta |  |
| IL2RA | NM_000417 | (CD25) Interleukin-2 receptor subunit alpha |  |
| IL6 | NM_000600 | Interleukin-6 |  |
| IL10 | NM_000572 | Interleukin-10 |  |
| IL15 | NM_000585 | Interleukin-15 |  |
| TGFB1 | NM_000660 | Transforming growth factor beta |  |
| TNF | [NM_000594](http://www.ncbi.nlm.nih.gov/entrez/query.fcgi?CMD=search&DB=gene&term=NM_000594&doptcmdl=Graphics) | Tumor Necrosis Factor – alpha (TNF-a) |  |
| TNFSF14 | NM_003807 | LIGHT/Tumor necrosis factor superfamily 14 |  |
| FOXP3 | NM_014009 | Forkhead box p3 | Innate immunity, Antigen recognition |
| AHR | NM_001621 | Aryl Hydrocarbon Receptor |  |
| CD3E | NM_000733 | CD3 - epsilon chain |  |
| CD74 | NM_004355 | HLA-DR antigens-associated invariant chain |  |
| TLR1 | NM_003263 | Toll-like receptor 1 |  |
| TLR2 | NM_003264 | Toll-like receptor 2 |  |
| TLR3 | NM_003265 | Toll-like receptor 3 |  |
| TLR4 | [NM_138554](http://www.ncbi.nlm.nih.gov/entrez/query.fcgi?CMD=search&DB=gene&term=NM_138554&doptcmdl=Graphics) | Toll-like receptor 4 |  |
| TLR5 | [NM_003268](http://www.ncbi.nlm.nih.gov/entrez/query.fcgi?CMD=search&DB=gene&term=NM_003268&doptcmdl=Graphics) | Toll-like receptor 5 |  |
| TLR7 | [NM_016562](http://www.ncbi.nlm.nih.gov/entrez/query.fcgi?CMD=search&DB=gene&term=NM_016562&doptcmdl=Graphics) | Toll-like receptor 7 |  |
| TLR8 | [NM_138636](http://www.ncbi.nlm.nih.gov/entrez/query.fcgi?CMD=search&DB=gene&term=NM_138636&doptcmdl=Graphics) | Toll-like receptor 8 |  |
| TLR9 | [NM_017442](http://www.ncbi.nlm.nih.gov/entrez/query.fcgi?CMD=search&DB=gene&term=NM_017442&doptcmdl=Graphics) | Toll-like receptor 9 |  |
| SLC9A1 | NM_003047 | Na+-H+ -exchange protein | Ion channel |
| GPBAR1 | NM_170699 | G protein-coupled bile acid receptor 1 (aka TGR5) | Ion/water secretion |
| GUCA2B | NM_007102 | Guanylate cyclase activator 2B (uroguanylin) |  |
| PDZD3 | NM_024791 | PDZ domain containing 3 |  |
| INADL | NM_176877 | InaD-like (Drosophila), PDZ activation |  |
| P2RY4 | NM_002565 | Pyrimidinergic receptor P2Y, G-protein coupled, 4 | Glial cell function |
| HAAO | NM_012205 | Kydroxyanthranilic acid oxygenase (3-hydroxyanthranilate 3,4-dioxygenase); synthesis of QUIN | activating NMDA receptors |
| RBP2 | NM_004164 | Retinol binding protein 2, cellular | Vitamin A absorption |
| ACTB | NM_001101 | Actin, beta | Housekeeping |
| GAPDH | NM_002046 | Glyceraldehyde-3-phosphate dehydrogenase (HKG) |  |
| B2M | NM_004048 | Beta 2 microglobulin (Housekeeping gene) |  |
| HGDC | SA_00105 | Human Genomic DNA Contamination | Assay CONTROL |

**Supplemental Table 2. Medication and Supplement Use in Participants**

**OPTION 1**

| **Category** | **Medication** | **Frequency** | **Healthy** | **IBS-D** | **IBS-C** |
| --- | --- | --- | --- | --- | --- |
| **No Medications** | |  | **3** | **3** | **0** |
| **Supplements** |  |  | **13** | **4** | **23** |
|  | Multivitamins | Daily | 5 | 0 | 7 |
|  | Iron | Daily | 1 | 1 | 0 |
|  | Fish oil | Daily | 3 | 0 | 2 |
|  | Probiotic | Daily | 1 | 0 | 0 |
|  | Calcium/ Vitamin D | Daily | 1 | 1 | 6 |
|  | Vitamin B12 | Daily | 0 | 1 | 2 |
|  | Glucoasmine-chondroitin | Daily | 2 | 0 | 1 |
|  | Fiber | Daily | 0 | 1 | 1 |
|  | Vitamin C | Daily | 0 | 0 | 2 |
|  | Black Cohosh | Daily | 0 | 0 | 1 |
|  | Vitamin E | Daily | 0 | 0 | 1 |
| **Allergy Medication** |  |  | **7** | **8** | **11** |
|  | Over the Counter, NOS | PRN | 1 | 0 | 0 |
|  | Loratadine | Daily | 1 | 0 | 2 |
|  | Cetirizine | PRN | 1 | 1 | 0 |
|  | Ranitidine | PRN | 0 | 1 | 0 |
|  | Fexofenadine/pseudoephedrine | Daily | 1 | 0 | 0 |
|  | Fexofenadine | Daily | 1 | 1 | 2 |
|  | Diphenhydramine | Daily | 1 | 1 | 3 |
|  | Fluticasone spray | Daily-BID  PRN | 1 | 2  1 | 4 |
|  | Hydroxyzine | PRN | 0 | 1 | 0 |
| **Asthma Medication** |  |  | **5** | **2** | **9** |
|  | Albuterol | PRN | 4 | 2 | 7 |
|  | Albuterol-ipratropium | PRN | 0 | 0 | 1 |
|  | Mometasone | PRN | 1 | 0 | 0 |
|  | Montelukast | Daily | 0 | 0 | 1 |
| **Analgesics/Salicylates** |  |  | **8** | **4** | **15** |
|  | Aspirin | Daily | 2 | 0 | 3 |
|  | Ibuprofen | As needed | 4 | 3 | 6 |
|  | Celecoxib |  |  |  | 1 |
|  | Acetaminophen | PRN | 2 | 1 | 5 |
| **Antidepressants** |  |  | **2** | **5** | **9** |
|  | Citalopram | Daily | 1 | 1 | 1 |
|  | Venlafaxine | daily | 1 |  |  |
|  | Buproprion | Daily |  | 2 | 2 |
|  | Fluoxetine | Daily |  | 1 | 1 |
|  | Sertraline | Daily | 0 | 1 | 1 |
|  | Escitalopram | Daily | 0 | 0 | 2 |
|  | Duloxetine | Daily | 0 | 0 | 1 |
|  | Paroxetine | Daily | 0 | 0 | 1 |
| **Benzodiazepines** |  |  | **0** | **2** | **1** |
|  | Clonazepam | Nightly | 0 | 1 | 0 |
|  | Lorazepam | PRN | 0 | 1 | 1 |
| **Antidiarrheals** |  |  | **0** | **3** | **0** |
|  | Loperamide | PRN | 0 | 3 |  |
| **Laxatives** |  |  | **0** | **0** | **5** |
|  | Bisacodyl | Daily | 0 | 0 | 1 |
|  | Colace | PRN | 0 | 0 | 1 |
|  | Senna | 3x/week | 0 | 0 | 1 |
|  | Miralax | Daily | 0 | 0 | 1 |
|  | Milk of Magnesia | Daily | 0 | 0 | 1 |
| **Anti-hypertensives** |  |  | **0** | **5** | **6** |
|  | Losartan | Daily | 0 | 2 | 1 |
|  | Indapamide | Daily | 0 | 1 | 0 |
|  | Lisinopril | Daily | 0 | 1 | 0 |
|  | Metoprolol | Daily | 0 | 1 | 0 |
|  | Atenolol | Daily | 0 | 0 | 1 |
|  | Diltiazem | Daily | 0 | 0 | 2 |
| **Statins** |  |  | **0** | **1** | **4** |
|  | Rosouvastatin | Daily | 0 | 1 | 0 |
|  | Simvastatin | Daily | 0 | 0 | 1 |
|  | Pravastatin | Daily | 0 | 0 | 2 |
|  | Atorvastatin | Daily | 0 | 0 | 1 |
| **Antacids** |  |  | **0** | **2** | **4** |
|  | Pantoprazole | Daily | 0 | 1 | 2 |
|  | Omperazole | Daily | 0 | 0 | 1 |
|  | Ranitidine | Daily | 0 | 1 |  |
|  | Calcium carbonate | PRN | 0 | 0 | 1 |
| **Birth Control/**  **Hormone replacement** |  |  | **2** | **3** | **6** |
|  | Mirena IUD |  | 1 |  | 2 |
|  | Nexplanon |  |  | 1 |  |
|  | Estradiol replacement (patch, cream_ | Biweekly | 1 |  | 2 |
|  | Anastrozole | Daily | 0 | 1 |  |
|  | Oral Contraceptive pill | Daily |  | 1 | 2 |
| **Stimulants** |  |  | **0** | **0** | **2** |
|  | Armodafinil | Daily | 0 | 0 | 1 |
|  | Methylphenidate | Daily | 0 | 0 | 1 |
| **Antivirals** |  |  | **2** | **1** | **3** |
|  | Acyclovir | Daily | 1 | 0 | 1 |
|  | Valacyclovir | Daily | 0 | 0 | 2 |
| **Miscellaneous** |  |  |  |  |  |
|  | Sumatriptan | PRN | 1 | 1 | 2 |
|  | Zolmitriptan | Daily | 0 | 0 | 1 |
|  | Metformin | Daily | 0 | 0 | 2 |
|  | Cyclobenzaprine | TID | 1 | 0 | 0 |
|  | Zolpidem | Daily | 1 | 0 | 1 |
|  | Guaifenesin | TID | 1 | 0 | 0 |
|  | Oxybutynin | TID | 0 | 1 | 0 |
|  | Pramipexole | Daily | 0 | 1 | 0 |
|  | Levothyroxine | Daily | 0 | 1 | 4 |
|  | Melatonin | Daily | 0 | 0 | 1 |
|  | Zaleplon | Daily | 0 | 0 | 1 |
|  | Nitroglycerin SL | PRM | 0 | 0 | 1 |
|  | Furosemide | Daily | 0 | 0 | 1 |
|  | Lamotrigine | Daily | 0 | 0 | 1 |
|  | Prednisone | BID | 0 | 0 | 1 |

**Supplemental Table 2. Medication and Supplement Use in Participants OPTION 2**

| **Category** | **Medication classes** | | **Frequency** | **Healthy** | **IBS-D** | **IBS-C** |
| --- | --- | --- | --- | --- | --- | --- |
| **No Medications** | | |  | **3** | **3** | **0** |
| **Supplements and Vitamins** | | | Daily | **13** | **4** | **23** |
| **Allergy Medications** | Mostly antihistamine or topical steroids | | Daily or PRN | **7** | **8** | **11** |
| **Asthma Medication** | Topical sympathomimetic anti-cholineric or anti-inflammatory | | Daily or  PRN | **5** | **2** | **9** |
| **Analgesics** | Salicylate, minor analgesics, NSAIDs | | Daily | **8** | **4** | **15** |
| **Anti-acid secretion: PPIs** | | | Daily | **0** | **2** | **4** |
| **Antidiarrheals** | loperamide | | PRN | **0** | **3** | **0** |
| **Laxatives** | Osmotic or stimulant | | Daily or PRN | **0** | **0** | **5** |
| **Statins** | | | Daily | **0** | **1** | **4** |
| **Anti-hypertensives** | ACE inhibitors, ARBs, calcium channel or beta-blockers | | Daily | **0** | **5** | **6** |
| **Antidepressants** | SSRI, SNRI, dopaminergic | | Daily | **2** | **5** | **9** |
| **Benzodiazepines** | | | Nightly, PRN | **0** | **2** | **1** |
| **CNS Stimulants** | Daily | | **0** | **0** | **2** |  |
| **Antidiarrheals** | loperamide | | PRN | **0** | **3** | **0** |
| **Laxatives** | Osmotic or stimulant | | Daily or PRN | **0** | **0** | **5** |
| **Birth Control/ Hormone replacement** oral or topical or intra-uterine | | | Daily/ IUD/ bi-weekly | **2** | **3** | **6** |
| **Antivirals** | | | Daily | **2** | **1** | **3** |
| **Miscellaneous** | |  |  |  |  |  |
|  | | Sumatriptan | PRN | 1 | 1 | 2 |
|  | | Zolmitriptan | Daily | 0 | 0 | 1 |
|  | | Metformin | Daily | 0 | 0 | 2 |
|  | | Cyclobenzaprine | TID | 1 | 0 | 0 |
|  | | Zolpidem | Daily | 1 | 0 | 1 |
|  | | Guaifenesin | TID | 1 | 0 | 0 |
|  | | Oxybutynin | TID | 0 | 1 | 0 |
|  | | Pramipexole | Daily | 0 | 1 | 0 |
|  | | Levothyroxine | Daily | 0 | 1 | 4 |
|  | | Melatonin | Daily | 0 | 0 | 1 |
|  | | Zaleplon | Daily | 0 | 0 | 1 |
|  | | Nitroglycerin SL | PRM | 0 | 0 | 1 |
|  | | Furosemide | Daily | 0 | 0 | 1 |
|  | | Lamotrigine | Daily | 0 | 0 | 1 |
|  | | Prednisone | BID | 0 | 0 | 1 |

**Supplemental Table 3. Allergies Listed in Medical Records**

| **Allergies** | **Healthy** | **IBS-D** | **IBS-C** |
| --- | --- | --- | --- |
| No known Allergies | 9 | 8 | 10 |
| Environmental (Cat dander, pollen, dust mites, wasp) | 3 | 1 | 3 |
| Topical (Adhesives, Latex, wool) | 1 | 3 | 5 |
| **NSAIDS (Total)**  Naproxen  Ibuprofen | **1**  1 (edema) | **1**  1(colitis) | **1**(SOB) |
| **Opiates (Total)**  Codeine  Morphine  Propoxyphene | **1**  1 | **1**  1 | **5**  3  1  1 |
| **Antibiotics (Total)**  Amoxicillin/clavulanate  Penicillin  Minocycline  Doxycycline  Nitrofurantoin  Ciprofloxacin  Levofloxacin | **2**  1  1 | **3**  1  2 | **6**  1  1  1  1  1  1 |
| Fluorescein | 1 | 0 | 0 |
| Prednisone | 0 | 0 | 1 |
| Zolpidem | 0 | 0 | 1 |
| Carbamazepine | 0 | 0 | 1 |
| Sulfa drugs | 1 | 1 | 0 |
